# Phytotoxic effects of cigarette smoke on indoor plant *Epipremnum aureum*: *in vivo* analysis using chlorophyll *a* fluorescence transients

**DOI:** 10.3389/fpls.2025.1595713

**Published:** 2025-07-22

**Authors:** Garishma Shah, Upma Bhatt, Hanwant Singh, Hari Dev Chaudhary, Vineet Soni

**Affiliations:** Plant Bioenergetics and Biotechnology Lab, Mohanlal Sukhadia University, Udaipur, Rajasthan, India

**Keywords:** cigarette smoke, chlorophyll fluorescence, pollution, particulate matter, photosynthesis, abbreviation cigarette smoke (CS), cigarette smoke concentration (CSC), particulate matter (PM)

## Abstract

Indoor air pollution from cigarette smoke, poses significant threats to plant growth and development. This study investigates the impact of cigarette smoke on the indoor plant species *Epipremnum aureum* (money plant), focusing on morphological, biochemical, and physiological aspects, with an emphasis on photosynthetic efficiency. Plants were exposed to varying concentrations of cigarette smoke (0, 2, 4, 6, 8, 10 cigarettes/day) for 15 days in controlled conditions. Key findings include a significant reduction in the mean surface area (MSA) of leaves. Chlorophyll *a* fluorescence (ChlF) analysis revealed impaired PSII photochemistry, as evidenced by reduced fluorescence yields and a significant decrease in fluorescence intensity at higher smoke exposures. Specific energy fluxes, including absorption energy (ABS/RC), trapped energy (TR_0_/RC), and electron transport (ET_0_/RC), showed notable reductions, while dissipated energy (DI_0_/RC) increased significantly. The performance indices PI_ABS_ and PI_CS_ decreased with increasing smoke concentration and exposure duration, indicating a decline in overall photosynthetic efficiency. Principal component analysis demonstrated a distinct separation in the physiological response of plants based on exposure levels, with the highest cigarette smoke concentrations causing the most significant disruptions. The lethal dose calculations (LD_50_ = 6.4 cigarettes/day; LD_90_ = 9.9 cigarettes/day) highlight the severity of cigarette smoke’s impact on *E. aureum*. These results underscore the detrimental effects of cigarette smoke on indoor plants, suggesting that such pollutants can significantly impair plant health and stress the importance of managing indoor air quality.

## 1 Introduction

Air pollution stands out as a paramount environmental challenge, posing significant threats to global well-being. An alarming 90% of the global population is exposed to contaminated air, resulting in approximately seven million deaths annually (Dávila-Román et al., 2021; Organization, W. H, 2018; Tarín-Carrasco et al., 2021; Zacarias and Grubert, 2021). This menace extends beyond human health, adversely impacting ecosystems and the ozone layer (de Vries, 2021; Liao et al., 2021; Rupakheti et al., 2021). The origins of air pollutants are diverse, stemming from unregulated urbanization, industrial emissions, fossil fuel consumption, and tobacco smoke (Aksoy et al., 2021). Indoor air pollution, an overlooked yet critical facet, proves to be even more hazardous, with data indicating pollution levels up to 100 times higher than outdoor air (Caselli et al., 2010; Mason et al., 2000; Wood et al., 2006). Elevated concentrations of indoor air pollutants pose severe risks to human and plant health (Brilli et al., 2018; Cetin et al., 2019). Notably, cigarette smoke in indoor environments emerges as a significant contributor, introducing a plethora of toxic compounds (Zai et al., 2012). The toll on human life is staggering, with over eight million global deaths attributed to smoke exposure, encompassing both active smoking and passive exposure (non-smokers) (Organization, W. H and others, 2015). Understanding the nature and cause of indoor pollutants, as well as distinguish them from outdoor pollutants, is essential for health risk assessment (Obore et al., 2020). It is also vital for setting regulatory strategy for indoor air quality (IAQ) and developing effective mitigation strategies to reduce human contact to air pollution, irrespective of its origin (Blount et al., 2021; Isinkaralar et al., 2024; Rajagopalan and Goodman, 2021).

Tobacco products, including cigarettes and water pipes, are major contributors to indoor air pollution. Cigarette smoke comprises two types: mainstream smoke (inhaled by the smoker) and side-stream smoke (emitted from the burning end). Environmental tobacco smoke is composed of approximately 85% side-stream smoke, which contains higher levels of toxic gases. Likewise, mainstream smoke consists of 92% gaseous elements and 8% tar. Side-stream smoke contains higher levels of toxic gases compared to mainstream smoke. Cigarette smoke consists of two phases: a tar (particulate) phase and a gas phase, which together deliver an estimated load of over 10^17^ and 10^15^ free radicals per puff, respectively, as determined through electron spin resonance (ESR) spectroscopy (Pryor and Stone, 1993). The gas phase includes carcinogens such as acrolein, acrylonitrile, hydrocyanic acid, acetaldehyde, nitrogen oxides, and carbon monoxide, and remains active for a shorter duration than the tar phase. Cigarette smoke is highly damaging to human health, causing antigenic, cytotoxic, mutagenic, and carcinogenic effects. Given that individuals spend more than 80% of their time indoors, vulnerable populations—such as children, the elderly, and plants used for air purification—are at heightened risk from indoor air pollutant (Berglund et al., 1992).

Indoor plants are negatively affected by cigarette smoke, absorbing various toxic elements and contaminants from the air (Forster et al., 2018; Rumchev et al., 2017). Despite prohibitions on cigarette use in many countries, smoking still occurs in enclosed spaces (Fix, 2019). Indoor plants serve as effective biomonitors, absorbing pollutants and helping to assess IAQ. Several biomonitoring studies have highlighted the capacity of different plant species to capture and reflect air quality indices, including those impacted by cigarette smoke (Kumakli et al., 2017; Vimercati et al., 2016). However, prolonged exposure to smoke can result in morphological and physiological damage, such as chlorosis, necrosis, leaf curling, and reduced growth in ornamental species (Benson et al., 2019; Gushit et al., 2022; Viana, 2011). The presence of toxic metals from tobacco smoke necessitates monitoring IAQ at its source. Indoor plants face stresses from IAQ, water availability and concentration of smoke, impacting their ability to absorb toxic metals. Research indicates that ornamental species growing in smoky areas may experience leaf degradation due to this absorption. PM also attaches to various organs of plants such as hairy leaves or bark (Hatami-Manesh et al., 2021) which produce ill effect in plant development. Because leaves are the organs with air entry and exit through their stomata, and they interact with air the most. Leaves, being the primary interface for gas exchange, play a critical role in pollutant uptake. The extent of toxic metal absorption is influenced by the physical and chemical properties of the metals, plant species, organ morphology, surface area, surface texture, environmental conditions, and exposure duration (Shahid et al., 2019). PM also accumulates on rough surfaces such as hairy leaves or bark, further contributing to physiological stress (Hatami-Manesh et al., 2021). However, the accumulation of toxins may vary on the basis of plant organ and species of plants (Gorena et al., 2020). The differences among tobacco products and plant species can provide insights into air pollution effects and help identify potential bio-monitor species. Their exposure to smoke can lead to leaf degradation and stress. Numerous plant studies have also been conducted on bio-monitoring of toxins from cigarette smoke (Kumakli et al., 2017; Vimercati et al., 2016), but the direct physiological effects of cigarette smoke exposure on indoor plants remain underexplored.

The present investigation aims to assess the effects of cigarette smoke on plants physiology and morphology of the indoor plant species *Epipremnum aureum* (money plant). This species is chosen for its widespread presence and heightened sensitivity, making it a frequent subject in experimental studies. The central hypothesis is that continuous and prolonged exposure to cigarette smoke leads to measurable and dose-dependent physiological and morphological stress in *E. aureum*. These stress responses introduce adverse effects like reduced photosynthetic efficiency, loss of chlorophyll content, leaf deformation, and signs of oxidative damage.

Chlorophyll *a* fluorescence (ChlF) is employed as a widely accepted and non-invasive technique to detect plant stress conditions, often integrated with other physiological and chemical variables (Miranda et al., 2020; Percival et al., 2006). This provides a quantitative measure of oxygenic photosynthesis (Ashraf et al., 2019). Exposure to CS disrupts photosynthesis through chemical interactions with proteins, leading to an escalation in reactive oxygen species generation (Dobaradaran et al., 2021; Yadav, 2010). These findings will provide a more precise understanding of how smoke pollutants affect plants growth and development.

## 2 Materials and methods

### 2.1 Plant material

*Epipremnum aureum* plants were purchase from a local nursery in Udaipur, India. The plant specimens were identified by Dr. Vineet Soni using morphological characteristics such as climbing habit, presence of aerial roots that adhere to surfaces, and leaf morphology, heart-shaped and irregularly pinnatifid in mature plants, entire in juvenile plants, and trailing stems. The identification was further confirmed using the taxonomic key described in Basu et al. (2014) and cross-referenced with regional floras such as Bennet et al. (1987) for Araceae species. After purchasing plant age was around five weeks old, plants were kept in growth chamber at 28 ± 3°C and 70% relative humidity under a 16:8 h photoperiod (day/night) for 24 h at Plant Bioenergetics and Biotechnology Laboratory, MLS University, Udaipur, India. Following acclimation, the plants were cultivated for an additional two weeks in an open greenhouse under natural light conditions with supplemental growth materials. The soil used had a composition of 40–50% clay, 25–30% silt, and 25–30% sand, with a pH level of 7.10 and an organic matter content of 0.64% (w/w). During this cultivation period, the average temperature ranged from 30–32°C with a 12 h light/12 h dark photoperiod. At the time of experimentation, plants were approximately 7 weeks old and had reached a height of about 15 cm, indicating juvenile developmental stage. During the experimental phase, plants were kept under controlled laboratory conditions at 28 ± 3°C temperature, 75% relative humidity, and a 16:8 h photoperiod (day/night). A fluorescent lamp emitting photosynthetically active radiation (400–700 nm) at an intensity of approximately 50 μmol m^-2^ s^-1^ was used as the light source. Five morphologically similar individuals were selected and used as biological replicates for each treatment.

### 2.2 Experiment setup

Six experimental wooden chambers were designed, each with a floor area of approximately 0.46 m² (5 square feet) and a height of 2 meters, resulting in an internal volume of approximately 0.92 m^3^ per chamber. Each chamber was maintained under standard temperature and pressure (STP) conditions with a controlled photoperiod of 16:8 h light/dark. Six different treatments were established based on daily cigarette smoke concentration (CSC/Day): 0 (control), 2, 4, 6, 8, and 10 cigarettes per day, respectively. Each treatment had five biological replicates.

The average distance between each plant and the cigarette smoke source (artificial pump) was consistently maintained at 0.91 meters (3 feet). A standardized cigarette brand (Gold Flake Kings, ~70 mm length) was used. Cigarette smoke was generated using a custom-made artificial smoking device (flow rate ~17.5 mL/min) that mimicked human puffing intervals, burning each cigarette over ~5–7 minutes, according to the assigned treatment concentration. Each cigarette was puffed once every 30 seconds for 6–7 minutes. Smoke was released into the chamber via a small vent to ensure uniform dispersion. The experiment lasted 15 days, and measurements were recorded every third day.

### 2.3 Measurement of morphological parameters

To assess specific morphological variability in *E. aureum* exposed to CSC, the mean surface area (MSA) and mean damage area (MDA) were determined using Fiji-Image J software, an open-source tool for advanced image processing and scientific analysis (https://imagej.net/Fiji). Seven images were captured for each plant at three-day intervals over a 15-day period using a Nikon D7500 DSLR camera (20.9 MP resolution) from a distance of 70 cm under diffused light conditions.

### 2.4 Measurement of chlorophyll *a* fluorescence

Chlorophyll *a* fluorescence (ChlF) was measured using the Handy PEA fluorimeter from Hansatech Instruments Ltd., England. Prior to measurement, plants were dark-adapted for 50–60 minutes at 26°C. The ChlF signals were analyzed with Biolyzer v.3.0.6 software, developed by the Laboratory of Bioenergetics at the University of Geneva, Switzerland. The experiments were conducted in five replicates and repeated three times to ensure accuracy. The JIP-test method was employed to calculate various phenomenological and biophysical parameters, quantifying the behaviors of photosystem I (PSI) and photosystem II (PSII). The polyphasic ChlF rise, known as the OJIP curve, provided valuable insights into photosynthetic fluxes, with numerous parameters derived from it (Strasser, 1992; Strasser et al., 2000). **Table 1** presents the definitions, formulas, and abbreviations for the JIP-test parameters used in this study.

**Table 1:** The JIP-test parameters, along with their respective abbreviations, formulas, and definitions, are presented.

BASIC PARAMETERS CALCULATED FROM THE EXTRACTED DATA	
F_O_≅F_50µs_or≅F_20µs_	Fluorescence when all PSII RCs are open (Tsimilli-Michael, 2020)
T_FM_=tF_MAX_, t for F_M_	Time (in ms) to reach maximal fluorescence F_M_ (Stirbet et al., 2018; Tsimilli-Michael, 2020)
F_M_	Maximal fluorescence, when all PSII RCs are closed (Stirbet et al., 2018; Tsimilli-Michael, 2020)
BIOPHYSICAL PARAMETERS DERIVED FROM THE BASIC PARAMETERS	
*Specific energy fluxes (per RC : QA-reducing PSII RC), in ms^-1^*	
ABS/RC= M_O_× (1/V_J_)× (1/φ_Po_)	Absorption flux (exciting PSII antenna Chl a molecules) per RC (also used as a unit-less measure of PSII apparent antenna size) (Strasserf and Srivastava, 1995; Stirbet et al., 2018; Tsimilli-Michael, 2020)
TR_O_/RC =M_O_×(1/V_J_)	Trapped energy flux (leading to Q_A_ reduction), per RC (Tsimilli-Michael, 2020)
ET_O_/RC =M_O_×(1/V_J_)× (1-V_J_)	Electron transport flux (further than Q_A_^-^), per RC (Tsimilli-Michael, 2020)
DIo/RC = ABS/RC– TRo/RC	Dissipated energy flux per RC (at t = 0) (Tsimilli-Michael, 2020)
*Phenomenological energy fluxes (per CS: QA-reducing PSII cross section), in ms^-1^*	
TR_O_/CS_M_=(F_v_/F_M_) (ABS/CS_M_)	Trapped energy flux (leading to Q_A_ reduction) per RC (Tsimilli-Michael, 2020)
ET_O_/CS_M_=(Fv/F_M_) (1 - V_J_) (ABS/CS_M_)	Electron transport flux (further than Q_A_^-^) per RC (Tsimilli-Michael, 2020)
DI_O_/CS_M_=(ABS/CS_O_) - (TR_O_/CSm)	Total energy dissipated per reaction center (RC) (Tsimilli-Michael, 2020)
ABS/CS_M_=≈ *F*_o_	Absorbed photon flux per excited PSII cross section at time zero (Tsimilli-Michael, 2020)
*Quantum yields and efficiencies*	
φ_Po≡_TR_0_/ABS=[1 -(F_O_/F_M_)]	Maximum quantum yield for primary photochemistry (Tsimilli-Michael, 2020)
φ_Eo_≡ET_0_/ABS=[1-(F_O_/F_M_)]×(1-V_J_)	Quantum yield for electron transport (ET) (Strasserf and Srivastava, 1995)
ψ_Eo_≡ET_0_/TR_0_=(1-V_J_)	Efficiency/probability that an electron moves further than Q_A_^-^ (Strasserf and Srivastava, 1995)
ϕDo= Fo/Fm	Quantum yield (at t = 0) of energy dissipation (Strasserf and Srivastava, 1995)
*Performance indexes*	
$PI_{ABS}=\frac{1-\left(F_{O}/F_{m}\right)}{M_{O}/V_{j}}\times \frac{F_{m}/F_{o}}{F_{O}}\times \frac{1-V_{j}}{V_{j}}$	PerformanceindexforenergyconservationfromphotonsabsorbedbyPSIIuntilthe reduction of intersystem electron acceptors (Strasserf and Srivastava, 1995>; Stirbet et al., 2018; Tsimilli-Michael, 2020)
$PI_{CS}=\frac{ABS}{CS}\;\times \;\frac{1-\left(F_{O}/F_{m}\right)}{M_{O}/V_{j}}\times \frac{F_{m}/F_{o}}{F_{O}}\times \frac{1-V_{j}}{V_{j}}$	Performance index on cross section basis (Strasserf and Srivastava, 1995; Stirbet et al., 2018; Tsimilli-Michael, 2020)

### 2.5 Measurement of chlorophyll content

The Arnon, (1949) method was applied to ascertain the total chlorophyll content in *E. aureum* plants under CS. Fresh leaves were crushed in 80% acetone, and the resulting extract was collected in a 3 mL vial. The extract was then subjected to cold centrifugation at 4°C and 5000 rpm for five minutes. For the quantification of chlorophyll *a* (chl a) and chlorophyll *b* (chl b), the supernatant obtained after centrifugation was analyzed using a spectrophotometer at wavelengths of 663 nm and 645 nm, respectively. The absorbance values obtained were then used in the following formula to estimate the total chlorophyll content:


$$Total\;chl.\;content\;\left(\frac{mg}{g}\right)=\left(20.2\times \;A_{663}+8.02\times \;A_{645}\right)\times \frac{\text{V}}{1000\times \text{W}}$$

Where A_663_ and A_645_ are the absorbance values at their respective wavelengths, V is the volume of the extract (in mL), and W is the weight of the plant material used for extraction (in g).

### 2.6 Statistical analysis

Statistical analyses were conducted using ANOVA with Tukey’s HSD test (p = 0.05) compare the effects of different cigarette smoke concentrations on plant physiological and morphological parameters. A significance level of *p* ≤ 0.05 was used to determine statistically meaningful differences among treatments. Before conducting ANOVA, assumptions of normality (Shapiro–Wilk test) and homogeneity of variance (Levene’s test) were confirmed. Each treatment consisted of five biological replicates (i.e., five individual plants per treatment), and measurements were taken from each replicate. The analysis was conducted using SPSS software (version 22.0). Only statistically significant differences (*p* ≤ 0.05) were represented in the figures. For the heat map, values were normalized and scaled from 1 to 100, with high values (100%) represented in brown, medium values (50%) in orange, and low values (1%) in yellow. Principal Component Analysis (PCA) was carried out using Origin Pro 2018 to uncover patterns and variations in the data, based on eigenvalue decomposition of the data correlation matrix. Correlations between parameters were assessed using a grid correlation matrix, with results visualized using a color scale from +1 to -1, facilitated by Python software (Gomez-Flores et al., 2023; Sangani et al., 2019). Probit analysis was performed with SPSS (version 22.0) to estimate the lethal doses (LD_50_ and LD_90_), In this study, mortality was defined as complete leaf necrosis (≥90% of leaf area affected), which was standardized across treatments. A Chi-square test was employed to compare mortality ratios between experimental and control groups at different concentrations, ensuring the coefficient of variance was less than 7%.

## 3 Results

The growth of *E. aureum* was significantly affected by the CS, which caused modulation of the plant’s photosynthetic process. To investigate this phenomenon, the current study explored the impact of CSC on various parameters on *E. aureum* including morphological parameters, chlorophyll fluorescence, specific energy fluxes, phenomenological energy fluxes, and performance indexes.

### 3.1 Morphological parameters

The mean surface area (MSA) of *E. aureum* showed a progressive and significant decrease with increasing concentrations of CS, as presented in **Table 2**. The MSA was measured at regular intervals over 15 days and expressed in cm^2^. On day 0, the initial MSA for all treatments was approximately similar (~10.4–10.7 cm^2^). However, by the 9^th^ day, plants treated with 10 CSC exhibited a drastic reduction in MSA to 1.56 ± 0.187 cm^2^, representing a nearly 85% decline compared to the control (10.656 ± 0.011 cm^2^). By the 15^th^ day, the MSA further decreased to 0.452 ± 0.168 cm^2^ under 10 CSC exposures.

**Table 2:** Change in leaf area define as a mean surface area with the increasing CSC with days with *X ±* S for five replicate measurements at a 95% level of confidence.

Mean Surface Area (cm^2^)						
CSC	Day 0	Day 3	Day 6	Day 9	Day 12	Day 15
0	10.556 ± 0.011^a^	10.55 ± 0.097^ab^	10.596 ± 0.095^b^	10.656 ± 0.011^c^	10.696 ± 0.184^a^	10.636 ± 0.128^c^
2	10.674 ± 0.137^a^	10.2 ± 0.137^abc^	10.2 ± 0.137^b^	10.2 ± 0.185^cd^	8.418 ± 0.307^a^	8.2 ± 0.137^c^
4	10.718 ± 0.378^a^	9.26 ± 0.470^b^	8.51 ± 0.503^ab^	7.9 ± 0.248^d^	6.946 ± 0.714^ab^	6.66 ± 0.750^c^
6	10.434 ± 0.470^a^	8.51 ± 0.750^b^	6.66 ± 0.776^ab^	5.86 ± 0.147^d^	4.032 ± 0.677^abc^	3.476 ± 0.355^cd^
8	10.43 ± 0.231^a^	6.66 ± 0.176^bc^	5.86 ± 0.677^ab^	4.032 ± 0.171^d^	2.954 ± 0.246^abc^	1.968± 0.430^cd^
10	10.764 ± 0.150^a^	6.66 ± 0.376^bc^	5.86 ± 0.472^ab^	1.56 ± 0.187^d^	1.56 ± 0.472^c^	0.452 ± 0.168^bc^

Different letters indicate a significant difference (*P ≤* 0.05).

**Figure 1:**
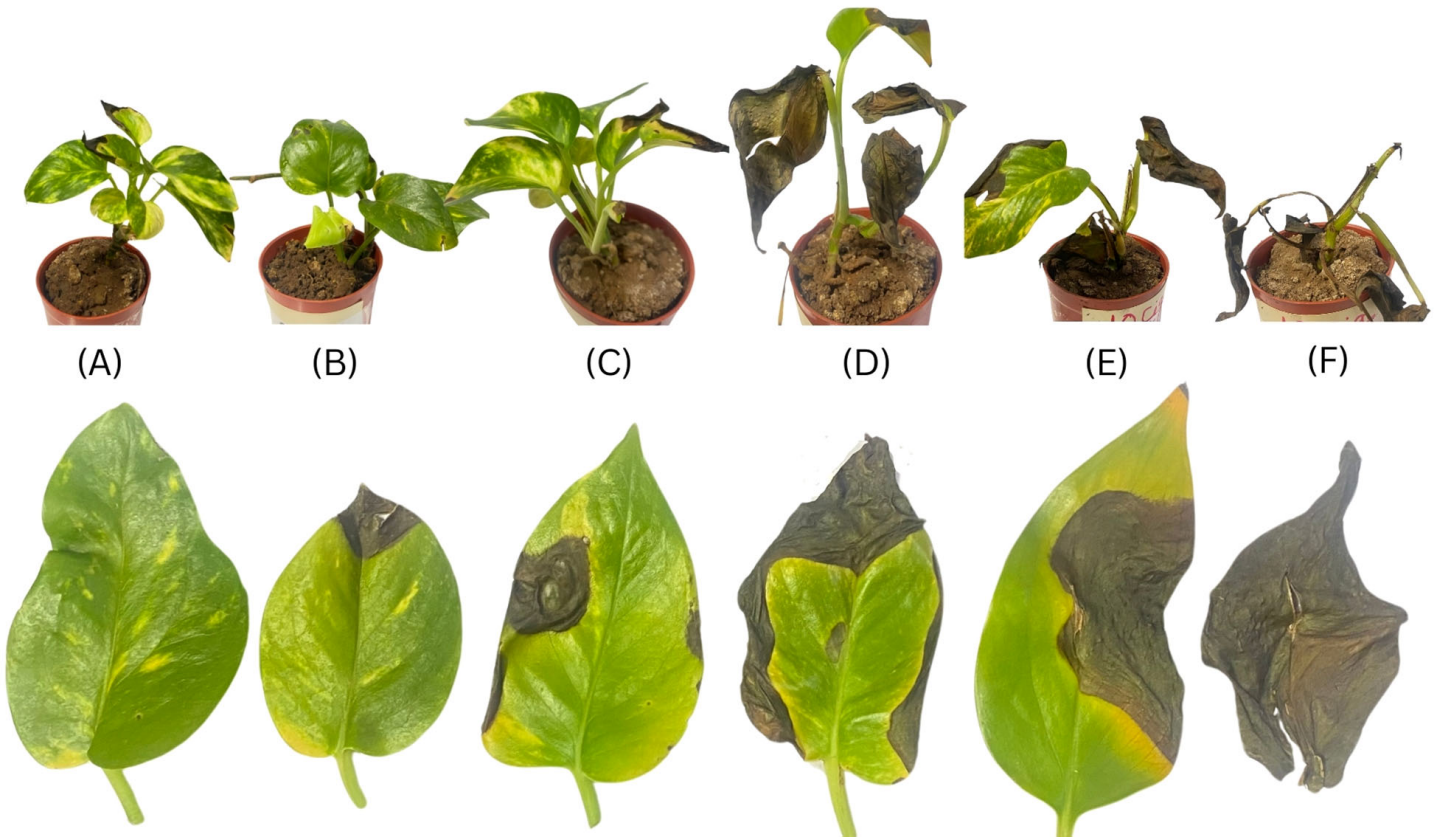
Progressive morphological deterioration in *E. aureum* exposed to increasing CSC over 15 days: Representative images of whole plants (top row) and corresponding leaves (bottom row) after 15 days of daily exposure to increasing CSC levels: **(A)** 0 CSC (control), **(B)** 2 CSC, **(C)** 4 CSC, **(D)** 6 CSC, **(E)** 8 CSC, and **(F)** 10 CSC. Plants were placed in a controlled exposure chamber and subjected to standardized CSC treatment daily. Visual symptoms include increasing chlorosis, necrosis, and tissue desiccation with higher CSC levels. Each treatment included 5 biological replicates (*n = 5*), and seven images were captured per treatment per day to document morphological responses. Shown here are representative samples from day 15. The severity of observed effects was consistent across replicates.

In contrast, the mean damage area (MDA) was calculated using mean surface area value as the difference between the initial surface area (day 0) and the final surface area (day 15). The MDA increased proportionally with CS concentration, indicating progressive leaf damage. The most extensive damage was observed in the 8 CSC and 10 CSC treatments, where the MDA reached 8.462 cm^2^ and 10.312 cm^2^, respectively. These results are visually represented in **Figure 1**, where leaves under 8 CSC (E) and 10 CSC (F) treatments show complete necrosis and collapse, correlating with the near-total reduction in leaf surface area. This confirms the severe phytotoxic effects of CSC at higher concentrations.

### 3.2 Total chlorophyll content

**Figure 2:**
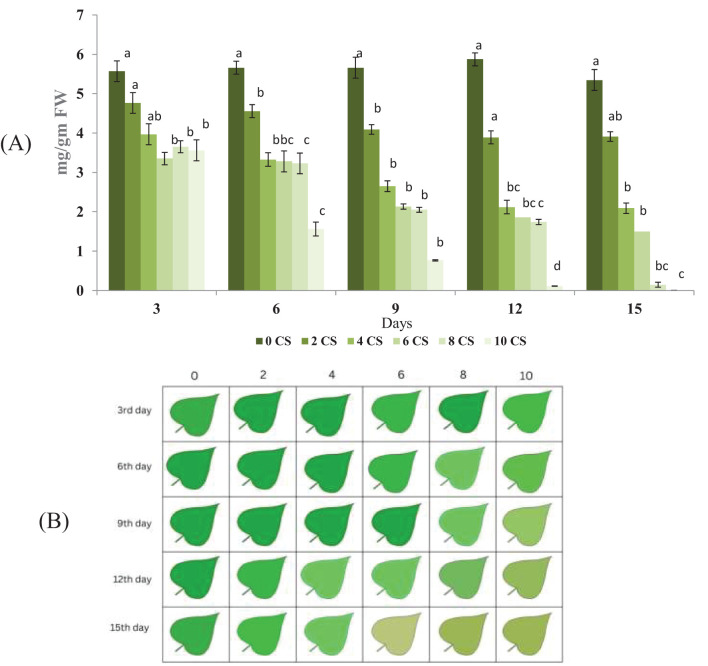
Chlorophyll content decline and visual color transition in *E.aureum* under increasing CSC over 15 days: **(A)** Total chlorophyll content (mg/g FW) measured in leaves of *E. aureum* exposed to 0, 2, 4, 6, 8, and 10 CSC for 15 days, recorded at 3-day intervals (Day 3, 6, 9, 12, 15). Chlorophyll estimation was performed using standard Arnon (1949) methods. Data represent mean ± SE of *n = 5*. Different lowercase letters above bars indicate statistically significant differences among treatments at each time point (ANOVA, *p*< 0.05, Tukey’s HSD). **(B)** Representative color-coded chlorophyll distribution patterns from Biolyzer software based on the same samples. Each cell represents the visual chlorophyll index of a single leaf under different CSC treatments (0–10) over five time points. A clear reduction in chlorophyll intensity and uniformity is visible with increasing CSC and duration of exposure.

The total chlorophyll content in plants over different exposure days under various CS treatments showed in **Figure 2B**. The control group consistently showed the highest chlorophyll levels across all days. As the duration increased, a gradual decline in chlorophyll content was observed in all treatment groups (3 to 15). This reduction was more pronounced in higher concentrations, particularly in 12^th^ and 15^th^. By the final day, 15^th^ showed the lowest chlorophyll levels, indicating severe degradation. The consistent downward trend highlights the negative impact of CS on chlorophyll synthesis. The data also reflect a time- and dose-dependent phytotoxic effect. Minimal error bars suggest reliable and reproducible measurements. Leaf impression showing green color variation estimate through biolyzer software present in **Figure 2A**. The green intensity of each leaf impression correlates with the estimated total chlorophyll level based on image fluorescence data.

### 3.3 Chlorophyll *a* fluorescence kinetics

**Figure 3:**
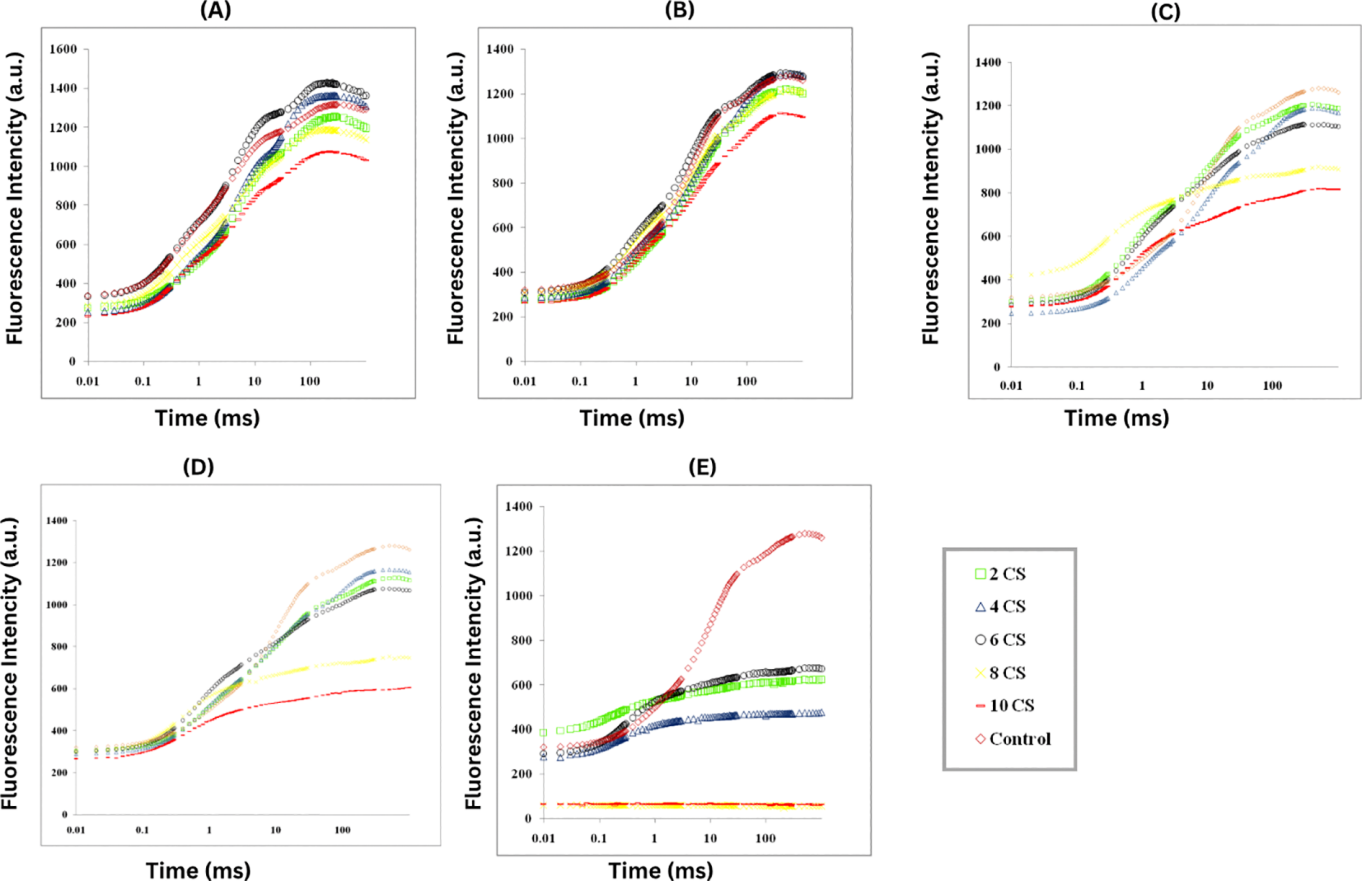
The study measured ChlF in *E. aureum* plants exposed to varying concentrations of CS (0-10) for 24 hours, using PSII rapid fluorescence transients (O, J, I, and P) as indicators **(A)** 3rd day **(B)** 6th day **(C)** 9th day **(D)** 12th day **(E)** 15th day. Fluorescence emission was measured from 10 µs to 1 s, capturing the characteristic O–J–I–P phases. Data represent the average (n = 5) with using one-way ANOVA followed by Tukey’s *post hoc* test (p< 0.05). Treatments not sharing the same letter indicate significant differences at the respective time points.

The chlorophyll fluorescence (ChlF) of *E. aureum* was assessed 3-days after exposure to CSC, revealing a typical OJIP induction curve when plotted on a logarithmic time scale. As the CSC increased, the fluorescence yield at J, I, and P steps were decreased. In control plants, two intermediate peaks, F_J_ (fluorescence intensity at 2 ms) and F_I_ (fluorescence intensity at 300 ms), were observed between F_0_ and F_M_. CSC exposure led to a reduction in PSII photochemistry and electron transport activity, especially at the highest CSC concentrations. Notably, on the 3^rd^ and 6^th^ days, the fluorescence intensity at 4 CSC and 6 CSC was higher than in the control conditions, but by the 9^th^, 12^th^, and 15^th^ days, the intensity had decreased to 25% of the control levels. Furthermore, after the 12^th^ day, the fluorescence intensity in plants exposed to 8 CSC and 10 CSC conditions had nearly diminished to zero. This fluorescent intensity is representing in **Figure 3** and **Supplementary Table 1**.

### 3.4 Biophysical parameters

**Figure 4:**
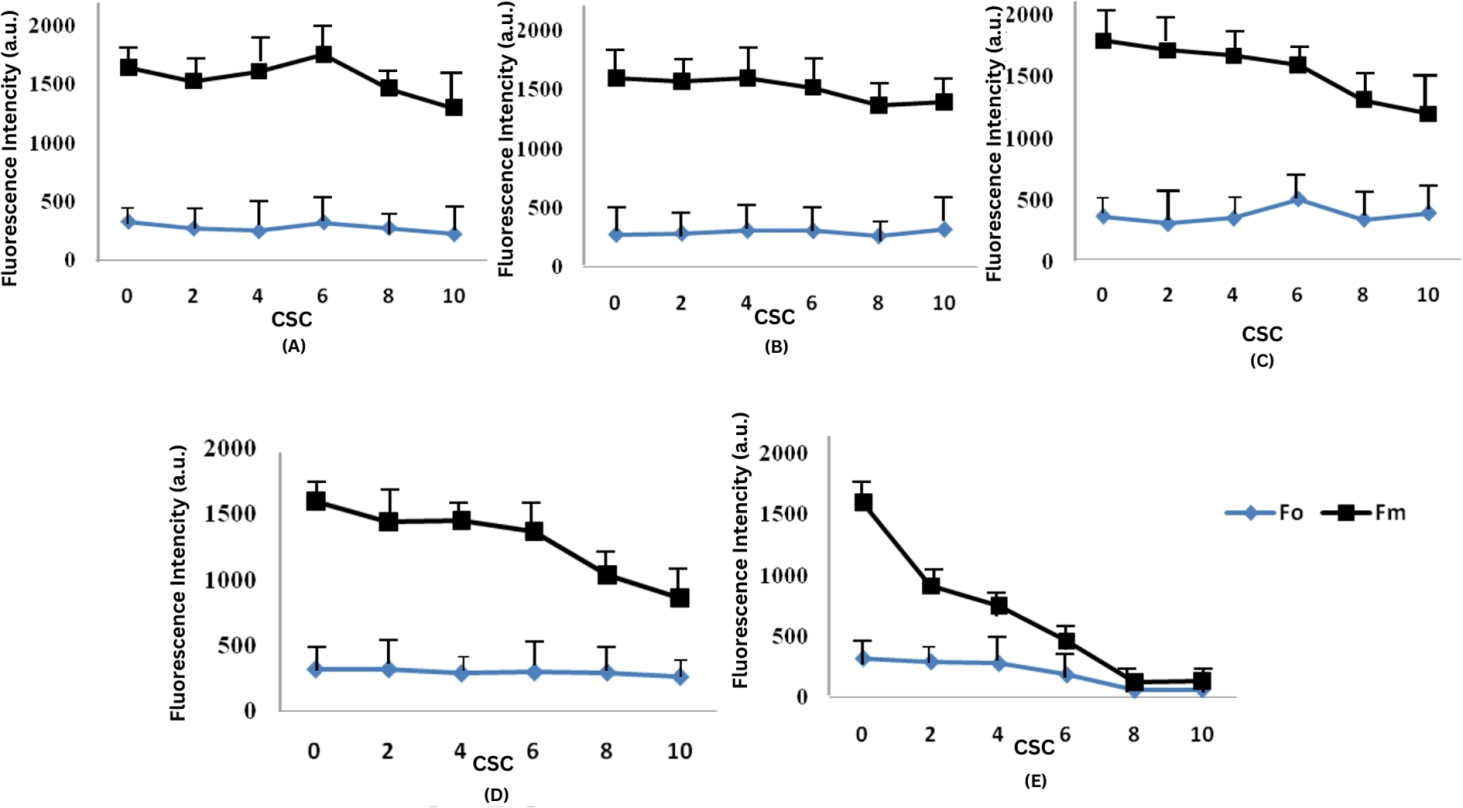
Effect of CSC on minimal (F_O_) and maximal (F_M_) chlorophyll fluorescence in *E. aureum*. The figure shows the changes in initial (F_0_) and maximum (F_m_) chlorophyll fluorescence intensity under varying concentrations of CSC: 0, 2, 4, 6, 8, and 10 CS over a 15-days **(A)** Day 3, **(B)** Day 6, **(C)** Day 9, **(D)** Day 12, and **(E)** Day 15. One-way ANOVA (n = 5). followed by Tukey’s *post hoc* test (p< 0.05).

CSC has been observed to reduce both the minimal fluorescence intensity (F_0_) and the maximal fluorescence intensity (F_M_). One-way ANOVA followed by Tukey’s HSD test (p< 0.05) confirmed statistically significant differences between treatment groups, particularly at higher CSC concentrations, indicating dose-dependent phytotoxic effects. F_0_ is the fluorescence measured at 50 μs when the primary quinone acceptor (Q_A_) is in its oxidized state. The efficiency of photosynthesis in plants is closely associated with the maximum primary yield of PSII photochemistry, as indicated in **Figure 4**. This ratio reflects the rates at which excited chlorophyll pigments undergo photochemical and non-photochemical deactivation. At concentrations of 4 CSC and 6 CSC, F_M_ values initially increased on the 6^th^ and 9^th^ days, but subsequently decreased to about 90% of the control values. In contrast, changes in F_0_ values were minimal.

### 3.5 Quantum yield

**Figure 5:**
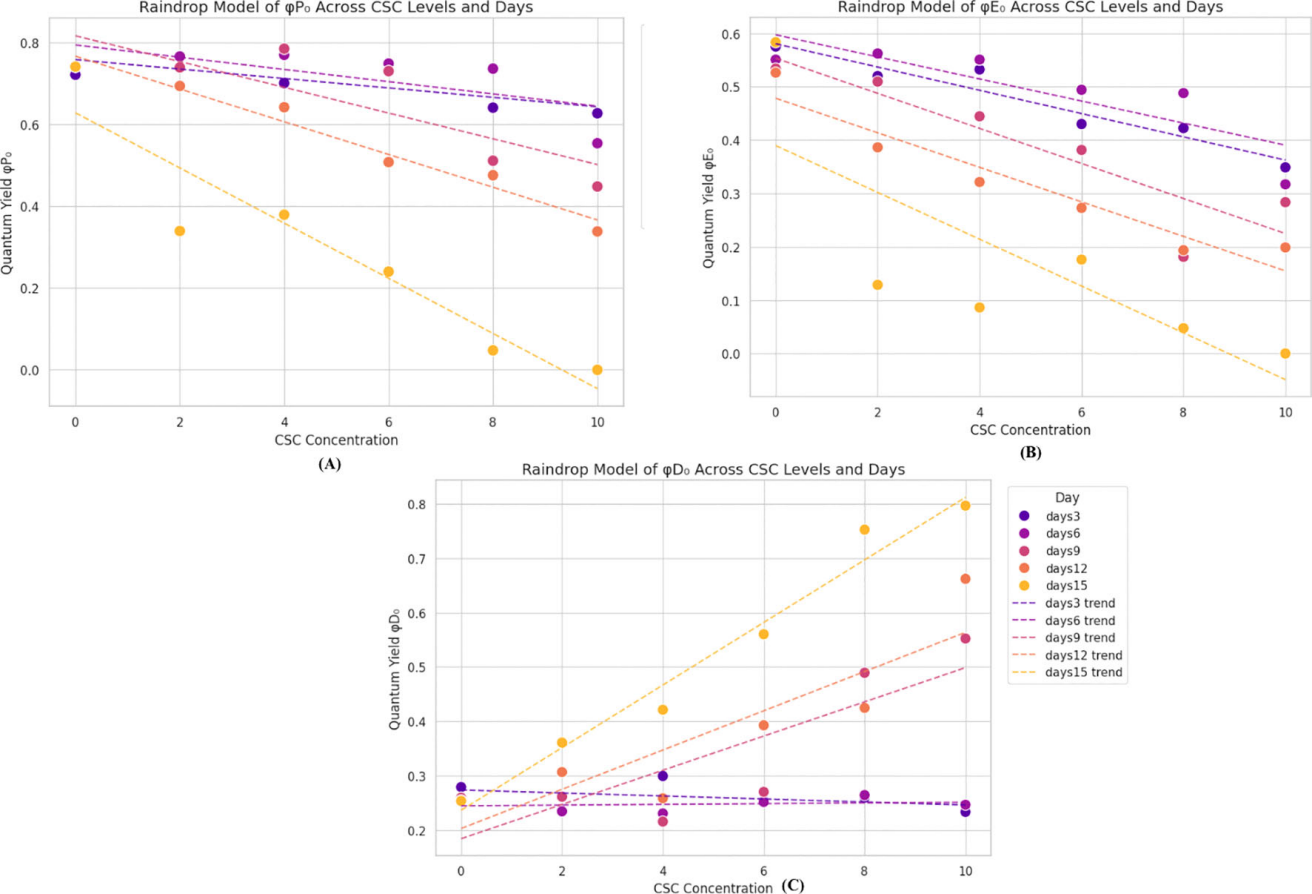
Raindrop regression models showing changes in chlorophyll fluorescence parameters in *E. aureum* exposed to increasing CSC concentrations (0–10) over 15 days. **(A)** φP_0_ (primary photochemistry) and **(B)** φE_0_ (electron transport) decreased with higher CSC and time. **(C)** φD_0_ (energy dissipation) increased, indicating enhanced protective response. Data represent means of five replicates (n = 5); trendlines show progression over time (Day 3–15). Significant differences were determined by two-way ANOVA with Tukey’s test (p< 0.05).

The introduction of CSC to plants led to a slight reduction in the quantum yield of primary photochemistry (φP_0_) and electron transport (φE_0_), φP_0_ reflects the efficiency of energy conversion at the primary photochemical step of PSII, while φE_0_ indicates the efficiency of electron transport beyond Q_A_^-^ both are indicators of the photosynthetic efficiency of active PSII RC This trend is evident in **Figure 5**. The minimal values of φP_0_ and φE_0_, approximately one fourth of the control, were recorded when *E. aureum* was exposed to 10 CSC. These values continuously decreased as the exposure duration and smoke concentration increased **Figures 5A–C**. In contrast, the quantum yield of dissipation (φD_0_) exhibited a continuous increase with rising CSC levels, showing an approximately two-fold increase compared to the control under the 10 CSC condition on the 4^th^ day. Furthermore, the φD_0_ value increased up to four times by the last day of the experiment under the 10 CSC condition. “These dynamics in energy fluxes are visually summarized in a ‘raindrop regression model’ diagram (**Figure 5**), where individual photochemical parameters (φP_0_, φE_0_, and φD_0_) are plotted over time and across CSC treatments, allowing for comparative visualization of their temporal and dose-dependent behavior. To support these visual interpretations, statistical analyses were performed using one-way ANOVA followed by Tukey’s *post hoc* test. The differences observed in φP_0_, φE_0_, and φD_0_ across treatment groups were found to be statistically significant (p< 0.05), confirming that the variations in quantum yields are not due to random chance but are a result of CSC exposure.

### 3.6 Specific energy fluxes

**Figure 6:**
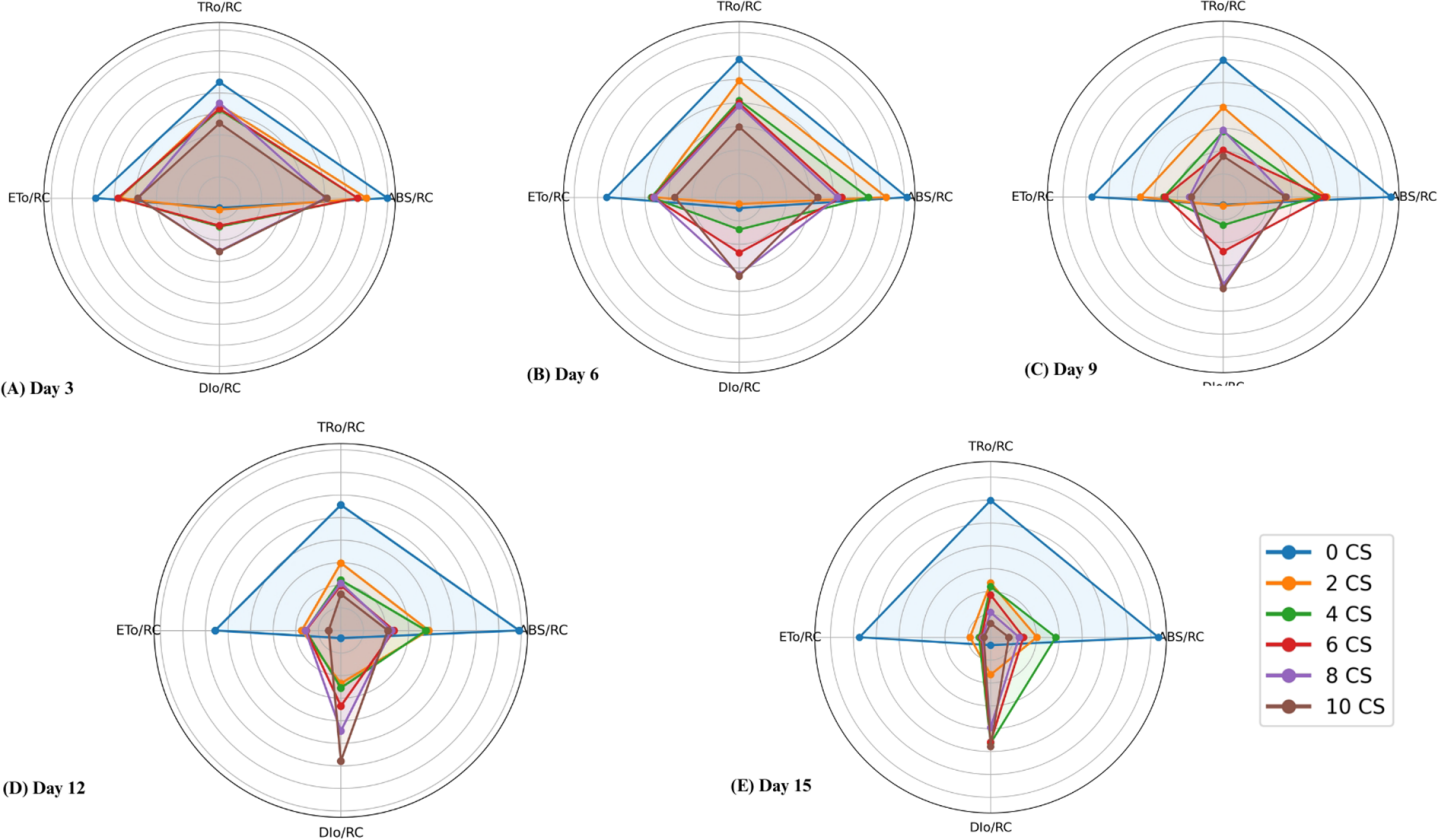
Radar plots illustrating specific energy fluxes per reaction center --ABS/RC (absorbed energy), TR_0_/RC (trapped energy), ET_0_/RC (electron transport), and DI_0_/RC (dissipated energy) in Epipremnum aureum leaves across different exposure durations: **(A)** Day 3, **(B)** Day 6, **(C)** Day 9, **(D)** Day 12, and **(E)** Day 15. As the concentration of cigarette smoke (CS) and exposure time increase, energy absorption, trapping, and electron transport (ABS/RC, TR_0_/RC, ET_0_/RC) decrease significantly, while energy dissipation (DI_0_/RC) increases sharply (p < 0.05).

The study analyzed the photosynthetic performance of active PSII RC in *E. aureum* under varying CSC by examining specific energy fluxes, including absorption energy (ABS/RC), trapped energy (TR_0_/RC), electron transport (ET_0_/RC), and dissipated energy (DI_0_/RC) flux per reaction center, as shown in area plot in **Figure 6**. The results indicated significant increase in ABS/RC at 4 and 6 CSC conditions up to the 9^th^ day, followed by a declining trend with increasing concentration and exposure duration. Minimal changes were observed in the values of TR_0_/RC and ET_0_/RC till the 9^th^ day, after which a gradual reduction was noted with increasing days and CSC. By the last day, TR_0_/RC and ET_0_/RC values had decreased by approximately 49.89% and 76.78% from the control, respectively, indicating a huge loss in energy trapping and electron transport efficiency. Conversely, DI_0_/RC displayed a substantial sequential increase, with approximately a fivefold increment observed in plants treated with 10 CSC compared to the control (**Figure 6**).

### 3.7 Phenomenological energy fluxes

**Figure 7:**
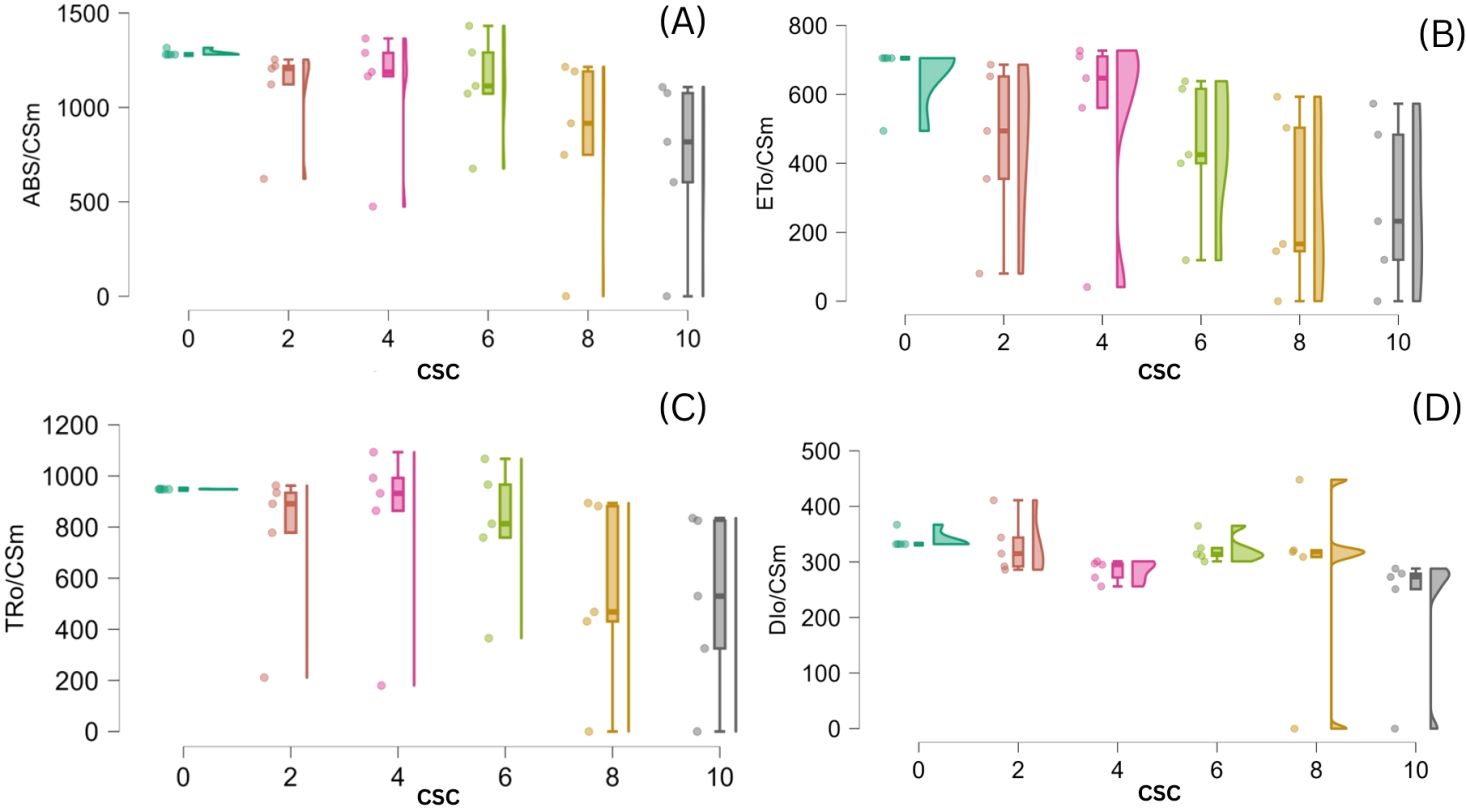
Violin (regression raindrop) plots depicting the effect of increasing cigarette smoke concentration (CSC) on phenomenological energy fluxes per cross-section in E. aureum leaves: **(A)** ABS/CSm, **(B)** ET_0_/CSm, **(C)** TR_0_/CSm, and **(D)** DI_0_/CSm. With rising CSC levels, absorbed, trapped, and transported energy fluxes (ABS/CSm, TR_0_/CSm, ET_0_/CSm) show a significant decline, while dissipated energy (DI_0_/CSm) increases, indicating impaired photosynthetic efficiency under cigarette smoke-induced stress (p < 0.05).

The impact of CSC-induced stress on *E. aureum* was assessed by examining changes in phenomenological energy fluxes, including absorption (ABS/CSm), trapped energy (TR/CSm), electron transport (ET/CSm), and dissipated energy (DI/CSm) flux per cross-section. Across all time points, increasing CSC concentrations led to a consistent and significant reduction in ABS/CSm, TRo/CSm, and ETo/CSm, with a concurrent increase in DIo/CSm. All these parameters exhibited significant reductions with increasing CSC levels in *E. aureum*. Specifically, under the 10 CSC condition on the 15^th^ day, ABS/CSm, TR/CSm, and ET/CSm, decreased by 50.13%, 75.89%, and 49.15%, respectively. Whereas DI/CSm increased by 82.60%, in the last day of CS exposure compared to the control represent in **Figure 7**. Statistical analysis (one-way ANOVA, p< 0.05) confirmed significant differences across CSC concentrations.

### 3.8 Performance index

**Figure 8:**
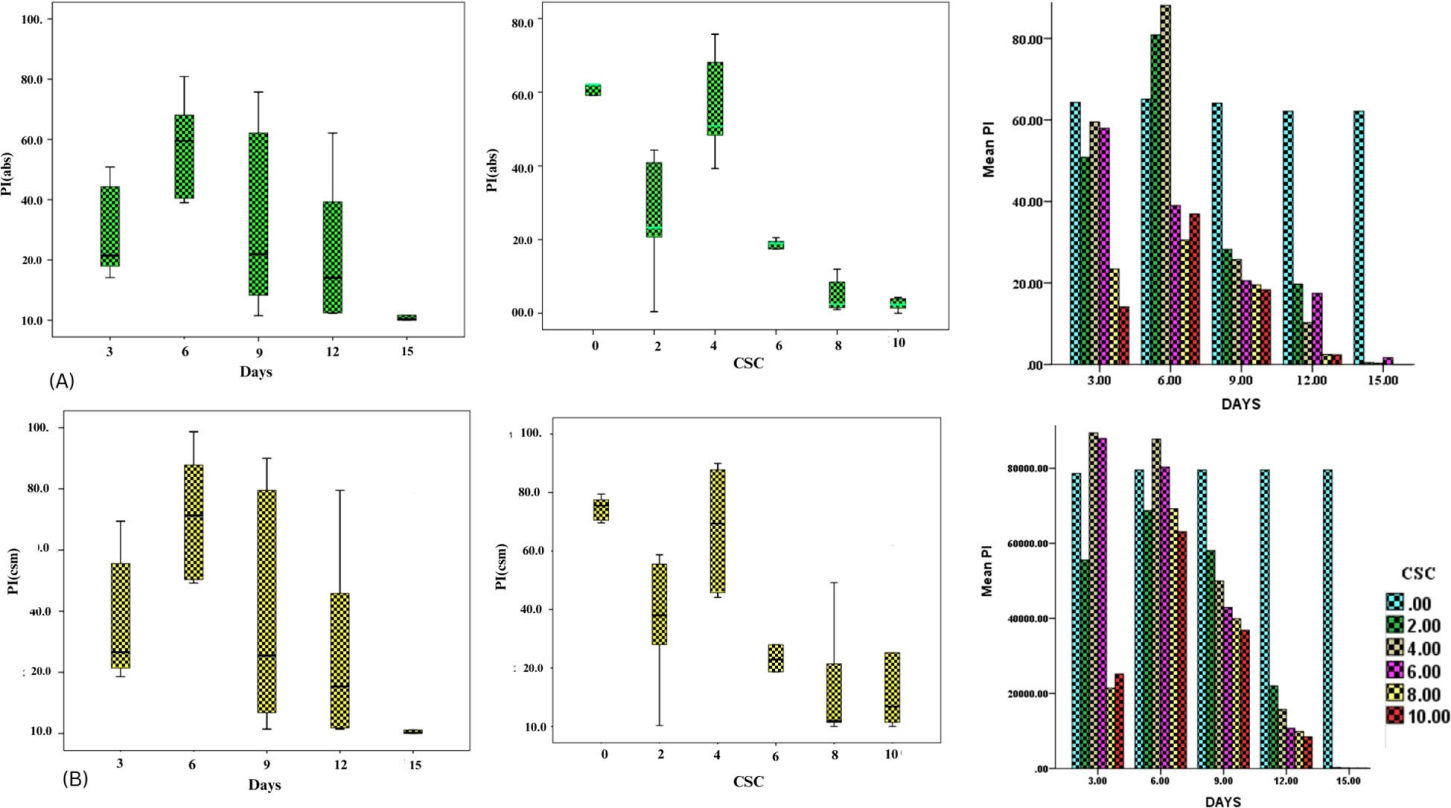
Box bar plot showing the PI_ABS_ and PI_CS_ **(A)** PI_ABS_ with (I) increasing concentration of CS and (II) increasing days (III) the both days and concentration with PI_ABS_ **(B)** PI_CS_ with (I) increasing concentration of CS and (II) increasing days (III) the both days and concentration with PI_CS_.

**Figure 9:**
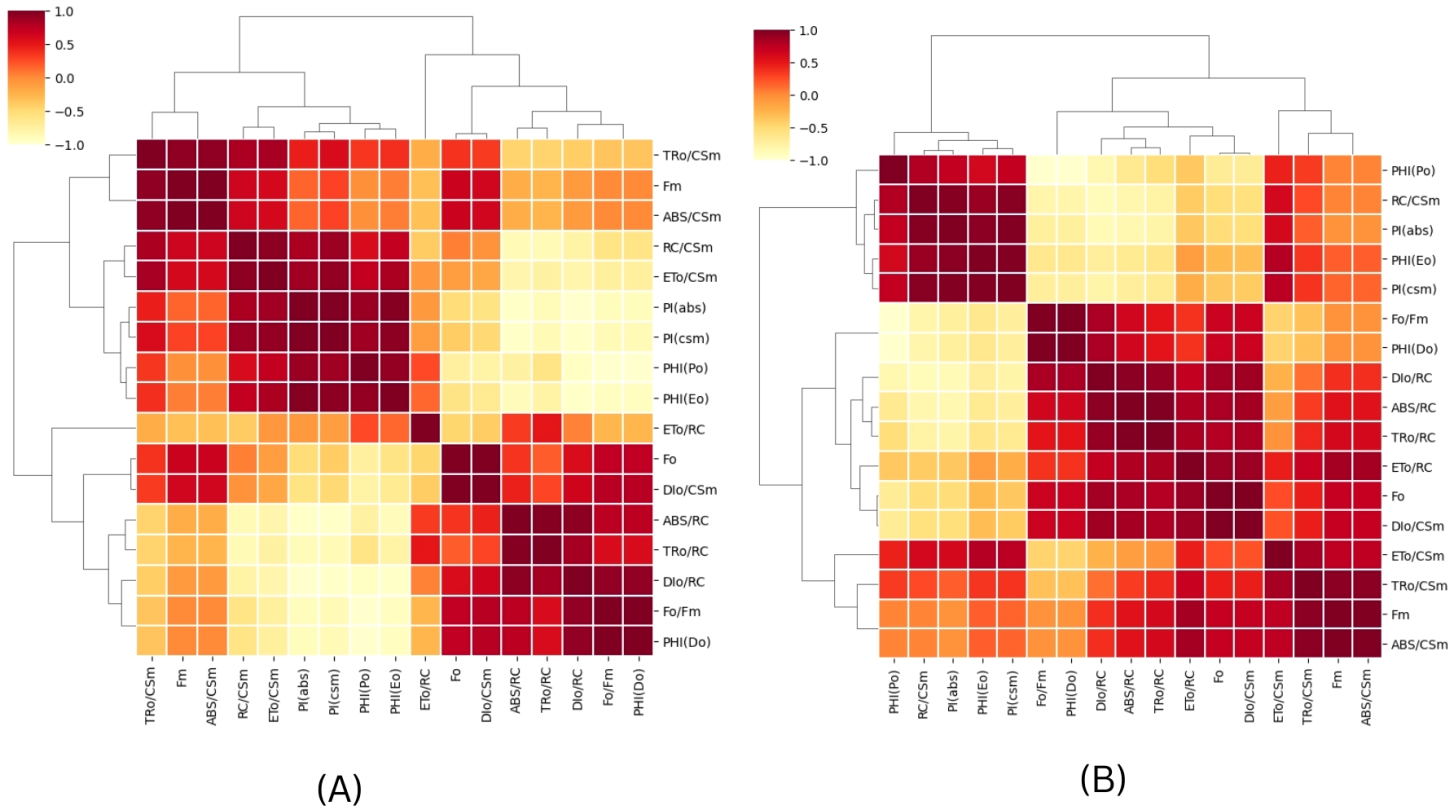
A Principal Component Analysis (PCA) was conducted using chlorophyll fluorescence data for **(A)** 3^rd^ and **(B)** 15^th^ day of CS exposure. The PCA generated two dimensions (PC1 and PC2), with PC2 capturing the majority of the variance in the data. The Chlorophyll a fluorescence parameter was represented by arrows on the PC1 and PC2 dimensions. All calculated chlorophyll *a* fluorescence parameters. The correlations were represented with a color code.

In order to assess the effects of CSC on the overall performance of photosynthesis, PI_ABS_ (performance index on absorption basis) and PI_CS_ (performance index on cross section basis) were measured in *E. aureum* plants subjected to different intensities of CSC stress. The results showed that CSC had a significant effect on PI_ABS_ and PI_CS_, with both parameters decreasing continuously as the CSC increased. In the box bar plot **Figure 8A**, PI_ABS_ and PI_CS_ were representing with the days. The toxicity of cigarette smoke increased over time, resulting in a progressive decline in photosynthetic performance, as reflected by reductions in both parameters (PI_ABS_ and PI_CS_) across the days. Along this the value of PI_ABS_ and PI_CS_ were 10 times lower compared to the initial day of exposure than the start day. In **Figure 8B**, PI_ABS_ and PI_CS_ were calculated on the axis of increasing smoke concentration, significant declining pattern were observed. Nevertheless in cluster bar graph **Figures 8A, B**, combine result was present with the time and CSC scale. The results of the PCA analysis showed that the first two principal components for day 3 **Figure 9A**, Dim 1 and Dim 2, explain 98.56% of the cumulative variation in the ChlF parameter under CSC induced stress in *E. aureum*. On day 3, the loading plot shows that several JIP-test parameters—including ET_0_/RC, ET_0_/CSm, PI_CS_, ABS/RC, TR_0_/RC, DI_0_/RC, TR_0_/CSm, ABS/CSm, DI_0_/CSm, φD_0_, F_O_, and F_M_—are grouped primarily in quadrant I. In contrast, performance indices and quantum yields such as PI_ABS_, φP_0_, and φE_0_ are located in quadrant II. Notably, the 10 CSC treatment group is distinctly separated from most JIP parameters, indicating reduced correlation under high CSC stress.

By the 15^th^ day (**Figure 9B**), PC1 and PC2 explain 98.28% of the cumulative variation. The distribution of JIP parameters remains largely consistent with day 3, still concentrated in quadrants I and II. However, unlike day 3, no chlorophyll fluorescence parameters are correlated with the 8 CSC and 10 CSC treatments, which are positioned distantly from the major clusters. This separation suggests a progressive decline or decoupling of these fluorescence responses under prolonged high CSC exposure. In quadrant IV, most of the parameters form obtuse angles with the origin, indicating negative or weak correlation with CSC treatments at this later stage.

**Figure 10:**
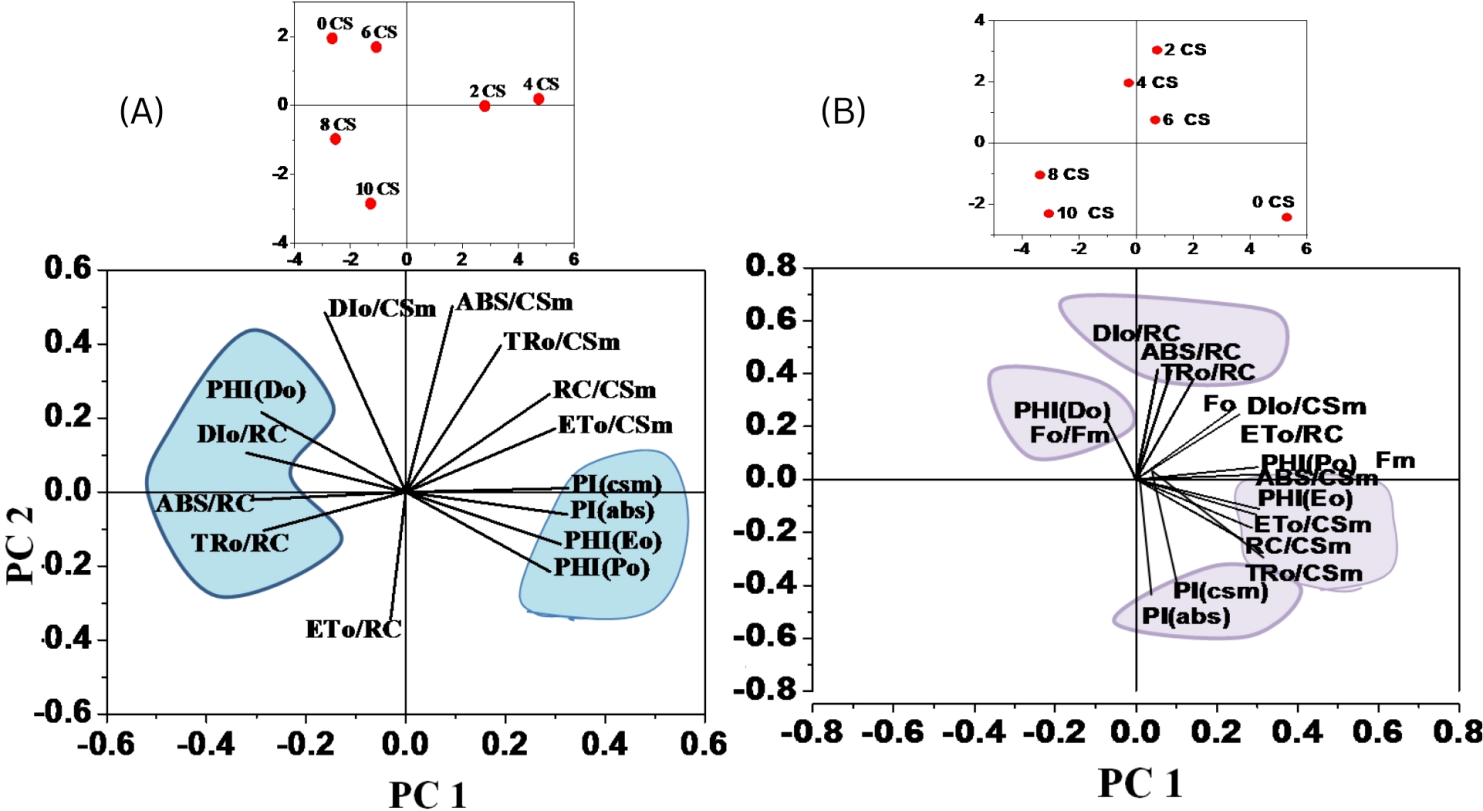
A heat map was used to illustrate the relative variability of multiple photosynthesis-related parameters obtained from the JIP test on *E. aureum* plants under CS stress. The data was collected for varying concentrations (0-10) after 24 hours, with orange indicating lower values (1%), yellow indicating medium (50%), and brown indicating the highest values (100%). Prior to color coding, all data was normalized to maintain unbiased results within a range of 1–100 for the parameter values. **(A)** relation after 3^rd^ day **(B)** relation after end say of experiment (15^th^ day).

Overall, the PCA shows strong clustering and consistent correlation among biophysical parameters, as confirmed by the correlation matrix (refer to **Figure 10**).

The LD_50_ and LD_90_ values for *E. aureum*, which were calculated by number of mortality rate through probit analysis with a 95% probability level displays in **Table 3**. LD_50_ and LD_90_ represent the lethal dose necessary to cause 50% and 90% mortality, respectively. The values obtained for LD_50_ is 6.4 CSC and LD_90_ is 9.9 CSC.

**Table 3:** The probit analysis was used to determine the acute 48-hour LD_50_ values of CS in *E. aureum* plants, along with their corresponding confidence limits.

Confidence Limits				
Probit		95% Confidence Limits for concentration		
		Estimate	Lower Bound	Upper Bound
PROBIT	.010	.306	-9.713	2.897
	.020	1.031	-7.845	3.386
	.030	1.491	-6.666	3.702
	.040	1.837	-5.783	3.943
	.050	2.118	-5.068	4.143
	.060	2.358	-4.462	4.315
	.070	2.568	-3.932	4.468
	.080	2.756	-3.460	4.608
	.090	2.927	-3.033	4.736
	.100	3.085	-2.641	4.856
	.150	3.736	-1.042	5.376
	.200	4.255	.194	5.824
	.250	4.699	1.216	6.246
	.300	5.098	2.092	6.667
	.350	5.468	2.858	7.104
	.400	5.819	3.532	7.571
	.450	6.159	4.129	8.078
	.500	6.493	4.658	8.634
	.550	6.827	5.132	9.247
	.600	7.167	5.562	9.921
	.650	7.518	5.960	10.663
	.700	7.887	6.339	11.486
	.750	8.287	6.713	12.410
	.800	8.731	7.097	13.471
	.850	9.249	7.514	14.737
	.900	9.901	8.007	16.363
	.910	10.058	8.123	16.759
	.920	10.230	8.247	17.191
	.930	10.418	8.381	17.668
	.940	10.628	8.530	18.201
	.950	10.867	8.698	18.812
	.960	11.149	8.893	19.532
	.970	11.495	9.130	20.420
	.980	11.955	9.441	21.604
	.990	12.680	9.924	23.477

The logarithm used in the analysis was base 10.

## 4 Discussion

The detrimental effects of CS on the growth and physiological health of indoor plants are well-documented, with the extent of impact varying by plant species. In this study, exposure to low concentrations of CS (2 CSC) led to a minor reduction in the surface area of *E. aureum* plants. However, at higher concentrations (≥6 CSC), a significant decrease in surface area was observed. Previous study, by Prasad et al. (2015), have similarly reported that lower concentrations of cigarette butts (CBs) produce less pronounced effects, while higher concentrations (greater than 20 CBs) inhibit plant growth. Additionally, increased mortality rates were observed over time with higher CSC treatments, accompanied by a reduction in leaf number. This phenomenon is attributed to excessive exposure to CSC, which negatively affects plant survival. Moreover, gases from cigarette smoke, such as sulfur dioxide (SO_2_), nitrogen dioxide (NO_2_), carbon radicals, ammonia, and volatile organic compounds, can be absorbed through the stomata of the leaves. These gases may damage the leaves and alter photosynthetic capacity, particularly at high concentrations (Permana et al., 2023).

Decreases in chlorophyll content can lead to a reduction in antenna size (Kirst et al., 2024; Martini et al., 2024). Strategic modulation of chlorophyll content or antenna size—such as reducing excess pigment to prevent light oversaturation under high light conditions—has been shown in some cases to enhance photosynthetic efficiency by minimizing energy loss and photo-inhibition. However, excessive reduction may impair light capture and thus negatively affect photosynthetic performance. At the canopy level, reduced leaf chlorophyll content can increase leaf transmittance, improve light distribution within the canopy, and thereby enhance canopy photosynthesis, as suggested by modeling studies (Song et al., 2021). Conversely, there are instances where chlorophyll-deficient mutants exhibit decreased photosynthetic rates. For example, the mutation of OsClpP6 impairs the photosynthetic apparatus and chloroplast development (Dong et al., 2013). Similarly, the loss of the transcription factor OsPIL1 leads to a decrease in chlorophyll content, accompanied by a reduction in plant surface area (Mao et al., 2023). In our study, cigarette smoke exposure significantly reduced chlorophyll content in *E. aureum*, which was associated with a decline in photosynthetic efficiency and visible morphological stress, indicating the detrimental impact of smoke-induced chlorophyll loss on overall plant health.

The minimal fluorescence intensity values are a key indicator of the extent of irreversible damage to PSII, particularly to the light-harvesting complex II (LHCII). Such damage can hinder electron transfer on the reduced side of PSII (Goltsev et al., 2016; Kalaji et al., 2014). The disruption of the electron transport chain can be attributed to the degradation of the LHCII oligomer. Additionally, the presence of heavy metal ions in CS can enhance the dissipation of excitation energy as heat, a process known as non-photochemical quenching (Janeczko et al., 2005). A reduction in minimal fluorescence under high concentrations of cigarette smoke stress may indicate reduced PSII efficiency, likely due to conformational changes in the D1 protein induced by CS stress. These changes can alter the properties of PSII electron acceptors (Kalaji et al., 2014). Moreover, the presence of particulate matter in CS can limit the formation of PSI, PSII, and LHC complexes in *E. aureum*. Permana et al. (2023) conducted an experiment using CS exposure exclusively on *Calathea makoyana*, Their study demonstrated that prolonged exposure to CS under enclosed conditions resulted in visible chlorosis, reduced leaf turgor, and significant decreases in chlorophyll content, suggesting that CS-induced oxidative stress impairs photosynthetic efficiency and overall plant health. The oxygen-evolving complex within the PSII complex is essential for catalyzing redox reactions and splitting water during photosynthesis (Ifuku et al., 2010). This process produces dioxygen and hydrogen ions, which are necessary for ATP phosphorylation (Paul et al., 2022). Given the disruption of the photosynthetic apparatus by CS, our findings suggest that cigarette smoke stress may increase the physiological demand for water in *E. aureum*, as the plant attempts to compensate for impaired energy production by enhancing water-dependent processes such as ATP synthesis and organic compound biosynthesis. Water is a crucial component in plant physiological processes, especially photosynthesis (Treesubsuntorn et al., 2021).

The F_0_/F_M_ ratio is an important parameter in the JIP test that indicates the efficiency of primary light energy conversion in the PSII reaction center (Baker et al., 2004; Kalaji et al., 2012; Schansker et al., 2005). It is commonly used as a stress indicator in various photosynthetic studies. This ratio depends on the fluorescence levels of F_0_ and F_M_, and any decrease in F_M_ can lead to a reduction in the F_0_/F_M_ ratio with increasing levels of CSC. PCA reveals a positive correlation between dissipation per reaction center (DT_0_/RC) and the F_0_/F_M_ ratio. This indicates that as photochemical efficiency declines under CS stress, plants increasingly rely on non-photochemical energy dissipation pathways to protect the remaining functional PSII units. Such an increase in energy dissipation, reflected by higher DI_0_/RC, has also been reported under various abiotic stresses, including heat, salinity, and drought, as a photoprotective mechanism (Kalaji et al., 2016). Therefore, the observed increase in both DI_0_/RC and F_0_/F_M_ under elevated CS concentrations suggests that CS imposes photoinhibitory pressure on PSII, and the plant attempts to mitigate this through increased energy dissipation. However, the observed increase in F_M_ values at early stages of 4–6 CSC treatments may appear contradictory. This anomaly could be due to transient photoprotective adjustments or measurement artifacts. The analysis of fluorescence transients, commonly known as the JIP test, in photosynthetic organisms exposed to abiotic stress, reveals a significant decrease in the φP_0_ value. This reduction in φP_0_ is due to a decline in the photochemical efficiency of PSII as a result of CS-induced stress. Under light conditions, the observed decrease in the maximum quantum yield of PSII (φP_0_) indicates that CS stress disrupts the redox reaction following Q_A_ and delays electron transport between Q_A-_ and Q_B_ (Schansker et al., 2005). However, φP_0_ alone provides only a partial understanding of PSII function. To more accurately assess the condition of the PSII acceptor side, additional parameters such as φE_0_ (quantum yield of electron transport beyond Q_A_^-^) and φD_0_ were also considered. In our study, φE_0_ showed significant reductions at higher CS concentrations and prolonged exposure times, supporting the notion that CS stress impairs the electron transport between Q_A_^-^ and Q_B._ Conversely, φD_0_ increased, suggesting enhanced energy dissipation mechanisms as a photoprotective response to CS stress. These combined changes indicate that CS negatively affects PSII function by disrupting the redox reactions at the acceptor side and enhancing energy dissipation to mitigate photodamage, as also supported by previous findings (Mykhaylenko and Zolotareva, 2017).

These fluorescence declines particularly in PI_ABS_ and φP_0_, are consistent with photoinhibition mechanisms that limit PSII efficiency under oxidative stress. Reactive oxygen species generated by CS exposure likely disrupt photosynthetic proteins and membranes, thereby reducing the plant’s energy conversion capacity. Integrating these observations with oxidative stress markers would provide further clarity on the damage pathways. The ABS/RC ratio, which is determined by the total number of photons absorbed by chlorophyll molecules across all active reaction centers (RC) divided by the total number of active reaction centers, reflects the proportion of active versus inactive RCs. An increase in the number of active RCs leads to a higher ABS/RC ratio. Conversely, the TR_0_/RC ratio measures the maximum rate at which excitons are captured by RC with a decrease in this ratio indicating a reduction in the population of the primary electron acceptor Q_A_. An increased DI_0_/RC ratio suggests a diminished amount of Q_A_ remaining in its reduced state. The analysis of specific energy flux reveals that various sites within PSII are sensitive to environmental stressors (Gautam et al., 2014; Zushi et al., 2012). The present study shows that with increasing concentrations of CS, the efficiency of electron trapping and transport in PSII declines, as active reaction RC are converted into inactive ones, thereby reducing the overall number of functional RCs. This decline in active RCs directly contributes to diminished photochemical efficiency and impaired electron transport. This decline is evident from the reductions in TR_0_/CSm and ET_0_/CSm values (Strasserf and Srivastava, 1995; Tsimilli-Michael, 2020; Zushi and Matsuzoe, 2017). A decrease in the ET_0_/RC ratio indicates a reduced capacity for electron transport, as the inactive reaction centers are less capable of re-oxidizing the reduced Q_A_ (Rapacz et al., 2015). The DI_0_/RC ratio, which represents the total dissipation of untrapped excitation energy per active RC, is influenced by the ratio of active to inactive RCs. Dissipation can occur through various pathways, including heat, fluorescence, and energy transfer to other systems. Despite variations in the active/inactive RC ratios, the DI_0_/RC ratio remains relatively stable due to the effective utilization of energy by the active RCs (Grieco et al., 2015; Heber et al., 2011). Our results indicate that at higher CS concentrations (6, 8, and 10) during days 9, 12, and 15, the DI_0_/RC ratio increases significantly. This suggests that under severe stress, the decline in active RCs surpasses the compensatory mechanisms, resulting in elevated energy dissipation per RC.

The phenomenological fluxes per cross-section—ABS/CSm, TR_0_/CSm, ET_0_/CSm, and DI_0_/CSm—offer insights into the behavior of the entire PSII population rather than individual RCs. Under CS stress, ABS/CSm and TR_0_/CSm declined, implying impaired light absorption and trapping capacity across the PSII antenna system. The reduction in ET_0_/CSm further supports the view that CS impairs the linear electron transport chain, compromising the energy transduction processes essential for ATP and NADPH production (Kalaji et al., 2014; Stirbet et al., 2018). In contrast, DI_0_/CSm increased with CS exposure, indicating a shift toward energy dissipation as heat or fluorescence, a typical response to protect the remaining functional PSII units from excess excitation pressure (Yusuf et al., 2010).

The performance index of a plant is a sensitive indicator of stress effects on its physiological components. This index is calculated using parameters based on energy absorption (PI_ABS_) and cross-section (PI_CS_), with PI_CS_ being dependent on phenomenological energy flux (Baker et al., 2004; Srivastava and Strasser, 1999). The performance index integrates multiple factors including the density of reaction centers, trapping efficiency, and electron transport efficiency, analogous to the Goldman equation (Goldman, 1943). In our study, exposure to CS significantly reduced the PI_ABS_ and PI_CS_ values in *E. aureum*. The decline in PI_ABS_ is linked to decreased activity of the RC, which in turn diminishes the overall efficiency of these centers (Kalaji et al., 2014, 2017; Kumar et al., 2020). The efficiency of electron trapping during the light reactions is crucial for plant productivity, as it directly impacts the plant’s ability to generate energy for growth and development (Sharma et al., 2023; Zhang et al., 2018). Through statistical analyses, including PCA and correlation matrices, we identified several JIP test parameters—such as ABS/CSm, TR_0_/CSm, ET_0_/CSm, φP_0_, PI_ABS_, and PI_CS_—that exhibited a dose-dependent response to CS stress. Additionally, probit analysis of the LD_50_ values revealed that CS is highly toxic to *E. aureum*. LD_50_ of approximately 6.49, indicating that moderate levels of cigarette smoke can cause lethal stress in 50% of *E. aureum* plants. Early effects begin at lower concentrations (LD_10_ ≈ 3.08), suggesting high sensitivity to smoke exposure. The steep rise in mortality beyond LD_50_ highlights a dose-dependent toxicity pattern. These findings emphasize the harmful impact of cigarette smoke on indoor plants even at sub-lethal levels.

## 5 Conclusion

In conclusion, this study reveals the detrimental effects of CS on *Epipremnum aureum*, a commonly used indoor plant, highlighting the broader implications for indoor air quality and plant health. Our findings indicate that exposure to CS induces significant morphological alterations, leaf damage, and reduced overall vigor. These physical changes are accompanied by a reduction in chlorophyll content, measured using the Arnon (1949) method, and a decrease in photosynthetic efficiency, as indicated by changes in chlorophyll fluorescence parameters analyzed using Biolyzer software. These are important indicators of the plant’s overall health and vitality. The research shows a clear dose-response relationship, with higher concentrations of CS causing more severe damage, ultimately leading to plant mortality. This underscores the severe impact of CS, not only on plant life but also on the effectiveness of indoor plants in air purification. The results emphasize the necessity for improved indoor air quality management, particularly in environments where cigarette smoking is prevalent. By documenting these adverse effects, this study highlights the importance of using indoor plants as bio-indicators for air pollution and advocates for policies and practices aimed at reducing indoor pollutant levels. The evidence presented highlights the urgent need for a more integrated approach to managing indoor air quality, emphasizing its impact on both plant health and human well-being. Although research on plant–smoke interactions remains limited, our study contributes valuable insights to this emerging field. The observed effects of CS on indoor vegetation underline the importance of implementing stricter air quality regulations in enclosed environments where smoking occurs. To build on these findings, future research should incorporate molecular, physiological, and biochemical approaches to deepen our understanding of how airborne pollutants affect plant systems.

## Data Availability

The original contributions presented in the study are included in the article/**Supplementary Material**. Further inquiries can be directed to the corresponding author.
