# Supplementary material for: Phytotoxic effects of cigarette smoke on indoor plant Epipremnum aureum: in vivo analysis using chlorophyll a fluorescence transients
Source: Front Plant Sci. 2025 Jul 22;16:1595713. doi: 10.3389/fpls.2025.1595713 (PMC12321795; doi:10.3389/fpls.2025.1595713)
Supplement: Supplementary file 1 [file Table1.docx]

Table showing the fluorescence value at F_O_, F_J_, F_I_, F_P_ at exposure CSC from day 3 to 15

| **CSC** | **Fo** | **F_J_** | **F_I_** | **Fp** |
| --- | --- | --- | --- | --- |
| **DAY 3** | | | | |
| 0 | 339 ± 12ᵃ | 822 ± 25ᵃ | 1318 ± 30ᵃ | 1293 ± 22ᵃ |
| 2 | 275 ± 10ᵇ | 602 ± 20ᵇ | 1254 ± 27ᵃᵇ | 1196 ± 24ᵇ |
| 4 | 253 ± 8ᶜ | 638 ± 18ᵇ | 1362 ± 35ᵃ | 1310 ± 26ᵃ |
| 6 | 336 ± 11ᵃ | 816 ± 23ᵃ | 1422 ± 31ᵃ | 1365 ± 29ᵃ |
| 8 | 286 ± 9ᵇ | 688 ± 19ᵇ | 1187 ± 22ᵇ | 1137 ± 21ᶜ |
| 10 | 232 ± 7ᶜ | 593 ± 15ᶜ | 1077 ± 20ᶜ | 1033 ± 19ᵈ |
| **DAY 6** | | | | |
| 0 | 320 ± 4.1ᵃ | 575 ± 6.2ᵃ | 1243 ± 9.5ᵇ | 1262 ± 10.1ᵇ |
| 2 | 276 ± 5.7ᵇ | 534 ± 7.1ᵇ | 1209 ± 11.4ᵇ | 1201 ± 12.6ᶜ |
| 4 | 288 ± 6.3ᵇ | 579 ± 6.8ᵃ | 1278 ± 8.9ᵃ | 1282 ± 11.3ᵃ |
| 6 | 311 ± 5.0ᵃ | 653 ± 7.6ᶜ | 1285 ± 9.2ᵃ | 1279 ± 10.7ᵃ |
| 8 | 309 ± 4.8ᵃ | 622 ± 6.7ᵃᵇ | 1199 ± 10.0ᵇ | 1208 ± 13.2ᶜ |
| 10 | 264 ± 5.5ᶜ | 535 ± 5.9ᵇ | 1102 ± 12.4ᶜ | 1096 ± 11.7ᵈ |
| **DAY 9** | | | | |
| 0 | 320 ± 4.3ᵃ | 575 ± 5.9ᵃ | 1266 ± 10.5ᵃ | 1223 ± 11.2ᵃ |
| 2 | 300 ± 5.2ᵇ | 712 ± 6.8ᶜ | 1201 ± 12.1ᵇ | 1187 ± 10.4ᵇ |
| 4 | 247 ± 6.1ᵈ | 541 ± 6.5ᵃ | 1185 ± 9.7ᵇ | 1168 ± 10.6ᵇ |
| 6 | 286 ± 5.5ᶜ | 689 ± 7.3ᶜ | 1114 ± 10.8ᶜ | 1105 ± 9.8ᶜ |
| 8 | 419 ± 5.9ᵉ | 750 ± 8.2ᵈ | 907 ± 11.5ᵈ | 909 ± 10.2ᵈ |
| 10 | 276 ± 4.7ᶜ | 586 ± 6.1ᵇ | 811 ± 9.9ᵉ | 818 ± 10.0ᵉ |
| **DAY 12** | | | | |
| 0 | 320 ± 4.0ᵃ | 572 ± 5.6ᵃ | 1236 ± 9.8ᵃ | 1272 ± 10.5ᵃ |
| 2 | 304 ± 5.2ᵇ | 596 ± 6.2ᵃ | 1112 ± 10.1ᵇ | 1115 ± 11.3ᵇ |
| 4 | 289 ± 4.6ᶜ | 604 ± 5.9ᵃ | 1160 ± 9.4ᵇ | 1155 ± 10.0ᵇ |
| 6 | 301 ± 5.4ᵇ | 673 ± 6.8ᶜ | 1071 ± 10.9ᶜ | 1068 ± 11.1ᶜ |
| 8 | 301 ± 4.9ᵇ | 604 ± 6.1ᵃ | 738 ± 11.0ᵈ | 746 ± 10.6ᵈ |
| 10 | 267 ± 5.1ᵈ | 484 ± 5.7ᵇ | 595 ± 12.2ᵉ | 605 ± 11.4ᵉ |
| **DAY 15** | | | | |
| 0 | 397 ± 5.4ᵃ | 566 ± 6.7ᵃ | 1245 ± 10.3ᵃ | 1234 ± 9.8ᵃ |
| 2 | 387 ± 6.2ᵇ | 542 ± 6.4ᵃ | 618 ± 9.5ᵇ | 623 ± 10.1ᵇ |
| 4 | 279 ± 4.7ᶜ | 434 ± 5.8ᵇ | 471 ± 8.9ᶜ | 478 ± 9.2ᶜ |
| 6 | 292 ± 5.0ᶜ | 557 ± 6.9ᵃ | 667 ± 9.7ᵇ | 674 ± 10.4ᵇ |
| 8 | 59 ± 3.1ᵈ | 60 ± 3.3ᶜ | 57 ± 2.9ᵈ | 58 ± 3.2ᵈ |
| 10 | 67 ± 3.5ᵈ | 68 ± 3.1ᶜ | 65 ± 3.4ᵈ | 65 ± 3.6ᵈ |
|  |  |  |  |  |
|  |  |  |  |  |
